# Performance of a Noninvasive Time-Harmonic Elastography Technique for Liver Fibrosis Evaluation Using Vibration Controlled Transient Elastography as Reference Method

**DOI:** 10.3390/diagnostics10090653

**Published:** 2020-08-31

**Authors:** Tudor Voicu Moga, Ioan Sporea, Raluca Lupușoru, Alina Popescu, Alexandru Popa, Simona Bota, Roxana Șirli, Mirela Danilă, Anton Schlesinger, Heiko Tzschätzsch

**Affiliations:** 1 Department of Gastroenterology and Hepatology, University of Medicine and Pharmacy “Victor Babeș”, 300041 Timișoara, Romania; moga.tudor@yahoo.com (T.V.M.); isporea@umft.ro (I.S.); raluca_lupusoru@yahoo.ro (R.L.); gastro.popa@gmail.com (A.P.); roxanasirli@gmail.com (R.Ș.); mireladanila@gmail.com (M.D.); 2 Department of Functional Science, University of Medicine and Pharmacy “Victor Babeș”, 300041 Timișoara, Romania; 3 Department of Internal Medicine and Gastroenterology, Hepatology, Endocrinology, Rheumatology and Nephrology and Emergency Medicine, Klinikum Klagenfurt am Wörthersee, 9020 Klagenfurt, Austria; bota_simona1982@yahoo.com; 4 GAMPT mbH and Institute of Medical Informatics, 06217 Merseburg, Germany; anton.schlesinger@gampt.de; 5 Department of Radiology, Charité–Universitätsmedizin, 10115 Berlin, Germany; heiko.tzschaetzsch@charite.de

**Keywords:** performance, time-harmonic elastography, liver fibrosis, vibration controlled transient elastography

## Abstract

Aim: This study aimed to evaluate the diagnosis performance of time-harmonic elastography (THE) technique in real life in assessing liver fibrosis, considering vibration-controlled transient elastography (VCTE) as a reference method. Material and Method: We prospectively evaluated outpatients from the gastroenterology department. Liver stiffness (LS) was measured by the THE system by dedicated operators, and by VCTE by experienced operators. The diagnostic accuracy of THE in staging liver fibrosis was assessed. We also performed an intra- and interobserver reproducibility sub-analysis on a sub-group of 27 subjects, where liver stiffness measurements (LSM) were performed by a novice, an elastography expert, and an ultrasound expert. Results: Of the 165 patients, using VCTE cut-off values, 49.6% were F0-F1, 15.7% were F2, 6.6% were F3, and 28.1% were F4. A direct, significant and strong correlation (r = 0.82) was observed between LSM assessed by VCTE and THE, *p* < 0.0001. The cut-off for ruling out liver cirrhosis (LC) by THE on our study group was <1.61 m/s (7.77 kPa)–AUROC = 0.90 [95% CI (0.82–0.93)], Se = 90.7%, Sp = 66.6%, PPV = 55.7%, NPV = 93.9%. The cut-off for ruling in LC by THE was >1.83 m/s (10 kPa)-AUROC = 0.90 [95% CI (0.82–0.93)], *p* < 0.0001, Se = 65.1%, Sp = 96.7%, PPV = 90.3%, NPV = 85.7%. The overall agreement between examiners was excellent: 0.94 (95% CI: 0.89–0.97); still, the ICCs were higher for the more experienced elastography examiner: 0.92 (95% CI: 0.82–0.96) vs. 0.94 (95% CI: 0.87–0.97) vs. 0.97 (95% CI: 0.95–0.99). Conclusions: THE is a feasible and reproducible elastography technique that can accurately rule in and rule out advanced liver disease.

## 1. Introduction

Chronic liver diseases are associated with the accumulation of extracellular matrix through the liver parenchyma, leading to hepatic fibrosis [1]. Liver tissue stiffness correlates directly with the severity of hepatic fibrosis, which is essential for diagnosis, prognosis, management, and follow-up of chronic liver diseases, thus, an accurate evaluation of liver stiffness is a useful tool in clinical practice [2]. The “gold standard” method used in the assessment of hepatic fibrosis is liver biopsy, a costly, invasive method with potential complications [3]. Therefore, several non-invasive techniques, biologic and elastographic (ultrasound-based or MRI-based) tests, have been developed in the last two decades and seem to be gradually substituting liver biopsy [4]. According to the European Federation of Societies for Ultrasound in Medicine and Biology (EFSUMB) [5] and the World Federation for Ultrasound in Medicine and Biology (WFUMB) guidelines [6], the ultrasound-based elastographic methods are divided into strain and shear waves elastography (SWE) techniques. The last category includes vibration-controlled transient elastography (VCTE), point shear wave elastography (pSWE), and two-dimensional shear wave elastography (2D-SWE) [5,6]. Transient elastography was the first technique used for the assessment of liver stiffness (LS). Although it has some limitations, (e.g., it cannot be used in patients with ascites, it lacks ultrasound B-mode visualization for guidance, it has lower performance in overweight subjects) it is largely accepted by international guidelines [7]. pSWE and 2D-SWE proved to be accurate, fast, and cost-efficient, but they display small elastograms, which represent only a fraction of the whole organ and have limited penetration depth, thus reducing the diagnostic precision of these elastography methods in the evaluation of diffuse liver disease (non-uniform distribution of fibrosis) [8].

Unlike classic ultrasound-based elastography techniques that use transient stimulation, a novel two-dimensional time-harmonic elastography (THE) technique was recently developed [9]. Using continuous multifrequency range shear waves generated by an external vibration device integrated into the patient bed, THE can obtain elastograms, covering the full ultrasound field of view at depths up to 13 cm underneath the body surface, in the same manner as MRI elastography [10].

The aim of this study was to evaluate the diagnostic performance of THE in real life in assessing liver fibrosis, considering vibration controlled transient elastography (VCTE) as a reference method.

## 2. Material and Methods

### 2.1. Study Design and Population

We prospectively evaluated patients who were referred for liver fibrosis assessment in the outpatient clinic in a gastroenterology and hepatology department as well as healthy volunteers (mainly students) during a four-month period (September 2018–March 2019). Patients referred for liver fibrosis evaluation had different stages of liver fibrosis and compensated liver cirrhosis. Exclusion criteria were pregnancy, age below 18 years, recent major surgery, patients with ascites (due to the limitation of VCTE), patients with elevated liver enzymes more than 5 times the normal value, presence of focal liver lesions, and patients who did not have reliable VCTE measurements.

Healthy subjects were defined as having normal ultrasound aspect of the liver, spleen, and biliary system, normal LS value by VCTE (<5.5 kPa) [5], and without any known liver disease (HBV, HCV, nonalcoholic or alcoholic fatty liver disease), cardiac failure, or diabetes mellitus. 

LS was measured by means of the THE system (GAMPT mbH, Merseburg, Germany), by dedicated operators (with specific training from the manufacturer) and by means of vibration-controlled transient elastography, using the M and XL-probe (FibroScan^®^, Echosens, Paris, France) [11], by experienced operators [12]. All the operators were blinded to clinical and biological data and to the other elastographic technique. In all patients, 10 valid LS measurements were obtained on the same day, both by THE and by TE. 

The study protocol was conducted according to the Helsinki Declaration after the approval by our institution’s Ethical Committee from the University of Medicine and Pharmacy “Victor Babes” Timisoara, number 19/08.10.2018 (approved on 8 October 2018). All the patients and volunteers gave their informed consent before taking part in the study. The study was conducted according to the Helsinki Declaration. 

### 2.2. Clinical Assessment

Clinical, laboratory, anthropometric, and demographic data were collected as well as the results of VCTE and THE measurements. Laboratory data (ALT, AST, GGT, ALP, thrombocytes) when available, were recorded. Body mass index (BMI) was calculated and alcohol intake was assessed using AUDIT-C score [13]. 

### 2.3. Time Harmonic Elastography Evaluation

The THE technique and principles were described in previous articles by Tzschätzsch et al. [9,14]. For LS evaluation by the THE system (GAMPT mbH, Merseburg, Germany) we performed 10 acquisitions for each patient. All patients were in fasting condition for more than 4 h. The patient’s position was supine with the right arm in maximum abduction.

**Figure 1:**
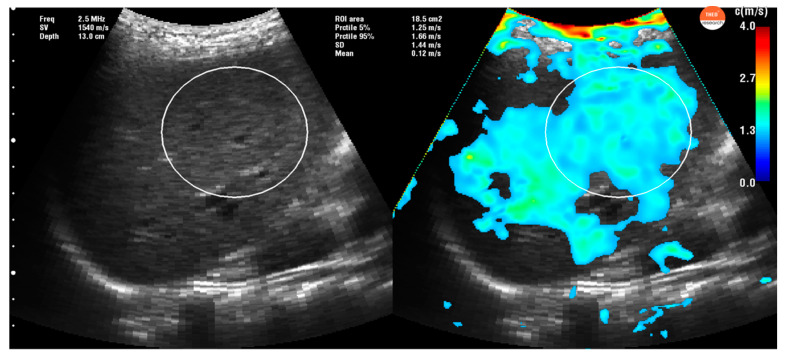
Liver stiffness measurement obtained with THE-Time Harmonic Elastography in a normal patient. In this picture, we can see the wide liver approach thru the intercostal section and the acquisition of the liver stiffness measurement (in the right side of the figure) reflecting healthy liver in cyan-green. Region of interest (ROI) is selected in the most homogeneous area. The current ROI has an area of 18.5 cm^2^ with a corresponding liver stiffness (mean ± SD, (5% percentile, 95% percentile)) of shear wave speed = 1.44 m/s ± 0.22 m/s (1.25 m/s, 1.66 m/s) and Young’s modulus = 6.22 kPa ± 1.90 kPa (4.69 kPa, 8.27 kPa), respectively.

**Figure 2:**
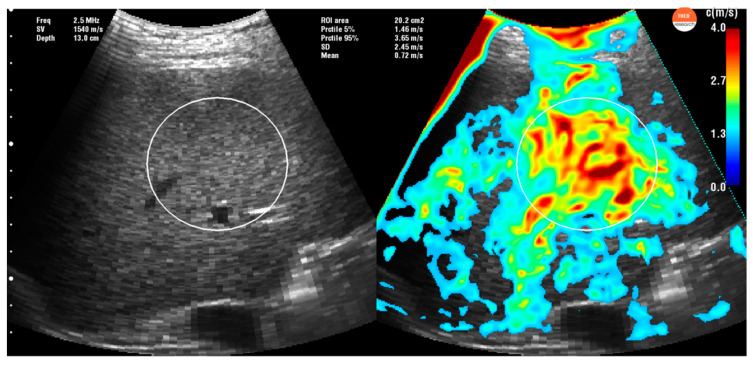
Liver stiffness measurement obtained with THE-Time Harmonic Elastography in a cirrhotic patient. In this picture, we can see the wide liver approach thru the intercostal section and the acquisition of the liver stiffness measurement (in the right side of the figure) reflecting cirrhotic liver in orange-red. ROI is selected in the most homogeneous area. The current region of interest has an area of 20.2 cm^2^ with a corresponding liver stiffness (mean ± SD, (5% percentile, 95% percentile)) of shear wave speed = 2.45 m/s ± 0.72 m/s (1.46 m/s, 3.65 m/s) and Young’s modulus = 18.01 kPa ± 10.58 kPa (6.39 kPa, 39.97 kPa), respectively.

A multifrequency time harmonic waveform was produced by a specially designed vibrator, fixed inside the examination bed that mechanically stimulates the liver at a standard of 27, 33, 39, 44, 50, and 56 Hz (GAMPT mbH, Merseburg, Germany). For B-mode–guided positioning of the elastography profile and acquisition of shear waves in the liver, an ultrasound probe (SonixMDP, Ultrasonix, Scottsdale, AZ, USA) with a 2.5 MHz transducer (C5-2/60 convex) was placed in the right intercostal spaces for a better liver approach. The patient was asked to hold apnea for a few seconds while the 1-second acquisition was being made. The postprocessing consists of (i) axial displacement estimation; (ii) harmonic frequency selection via temporal Fourier decomposition; (iii) spatial band pass filtering; (iv) spatial-directional filtering; (v) shear wave speed estimation via phase gradient method, and (vi) weighed averaging to calculate the final shear wave speed map (details can be found in [14]). An ROI (region of interest) was then selected on the most homogeneous area (Figure 1 and Figure 2) in each of the 10 measurements.

The mean, standard deviation, median value, and IQR (interquartile range) of THE LS measurements were calculated for each subject. No quality criteria have been described yet, thus we used the recommendation for all elastographic technique: IQR/med < 30% [14,15,16]. 

The THE results (shear wave speed-SWS) were expressed in m/s, but for comparison with VCTE we converted the values to Young’s modulus E (in kPa): (E = 3 × ρ × SWS^2^). Tissue density ρ is assumed to be 1000 kg/m^3^.

### 2.4. Vibration-Controlled Transient Elastography (VCTE)

VCTE was performed with FibroScan^®^ device (EchoSens, Paris, France) in fasting conditions for more than 4 h, by dedicated experienced examiners (more than 500 examinations) in the same day as THE technique. In each patient, we aimed for 10 valid LS measurements. The examination was performed in a supine position, by intercostal approach, with the right arm in maximum abduction, using the M probe (standard probe–transducer frequency 3.5 MHz) or the XL probe (transducer frequency 2.5 MHz). Both M and XL probes have been used according to the patients’ body mass index and according to the European recommendation on M and XL probe selection [9]. When pressing the transducer button, a mechanical vibrator mounted on the axis of the device generates a train of elastic waves that are transmitted into the liver. In parallel to the vibration, the transducer performs ultrasound acquisitions. By comparing the ultrasonographic signals thus obtained, tissue deformation records, induced by the propagation of the elastic wave, can be drawn. The time necessary for the train of waves to propagate along the interest area, as well as the velocity of propagation, is recorded. The liver stiffness can afterwards be calculated using the formula: E = 3 × ρ × Vs^2^ (E, the elasticity module; ρ, density; Vs, the elastic wave velocity in the liver parenchyma). The stiffer the tissue, the higher the velocity of the wave train. The software of the equipment analyzes the tissue deformation records and measures the stiffness of the parenchyma. The results are expressed in kilopascals (kPa) and represent the median value of 10 valid measurements. The equipment can measure values ranging between 2.5 and 75 kPa. Interquartile range/median (IQR/M) ratio < 30% was used as quality criteria [16,17]. The cut-offs taken in consideration to discriminate among different stages of as reported by Tsochatzis [18] were: 7.0 kPa for F ≥ 2, 9.5 kPa for F ≥ 3 and 12.0 kPa for F = 4. 

### 2.5. Inter and Intra-Reproducibility Assessment

For intra- and interobserver reproducibility sub-analysis, we used a sub-group of 27 subjects. We defined the examiner experience as follows: novice—no experience in elastography and less than 50 ultrasound examinations, elastography expert—more than one-year elastographic experience in VCTE, point shear wave elastography (pSWE) and 2D-SWE methods and ultrasound expert—more than 1000 performed examinations, without any experience in elastography. Through this sub-analysis, we wanted to reveal if elastographic or ultrasound experience plays a role in achieving reliable results with THE method, recalling that none of the THE operators had any experience with the machine apart from the minimum training from the manufacturer. 

### 2.6. Statistical Analysis

Statistical tests were performed using R software V.2.5.1 (R Development Core Team, Vienna, Austria) and IBM SPSS Statistics V.17 (IBM Statistics, Chicago, IL, USA). The Kolmogorov–Smirnov test was used for testing the distribution of numerical variables. Qualitative variables were presented as numbers and percentages. Parametric tests (*t*-test, ANOVA) were used for the assessment of differences between numerical variables with normal distribution; and nonparametric tests (Mann–Whitney or Kruskal–Wallis tests) for variables with non-normal distribution. The Chi-squared (χ^2^) test was used for comparing proportions expressed as percentages (“*n*” designates the total number of patients included in a particular subgroup).

Areas under receiver operating characteristic (AUROC) curves were calculated for THE values to identify discriminating cut-offs for various stages of liver fibrosis. The optimal cut-off values were determined from AUROC curve analysis, by using the Bayesian analysis, using the optimal criterion that takes into account the disease prevalence and the costs of false positive and false negative and sensibility/specificity greater than 90%, depending on what we wanted [rule in or rule out advance liver disease (ALD)]. Positive predictive value (PPV) and negative predictive value (NPV) were calculated. Univariate regression analysis and multivariate regression analysis were used in order to identify the factors associated with THE values. 

We used the intraclass correlation coefficient (ICC) and inter-rater agreement (Kappa coefficient) and concordance correlation coefficient to assess the intra- and interobserver reproducibility of THE system. 95% confidence intervals were calculated for each predictive test and a *p*-value < 0.05 was considered significant for all statistical tests.

## 3. Results

### 3.1. Baseline Characteristics

**Figure 3:**
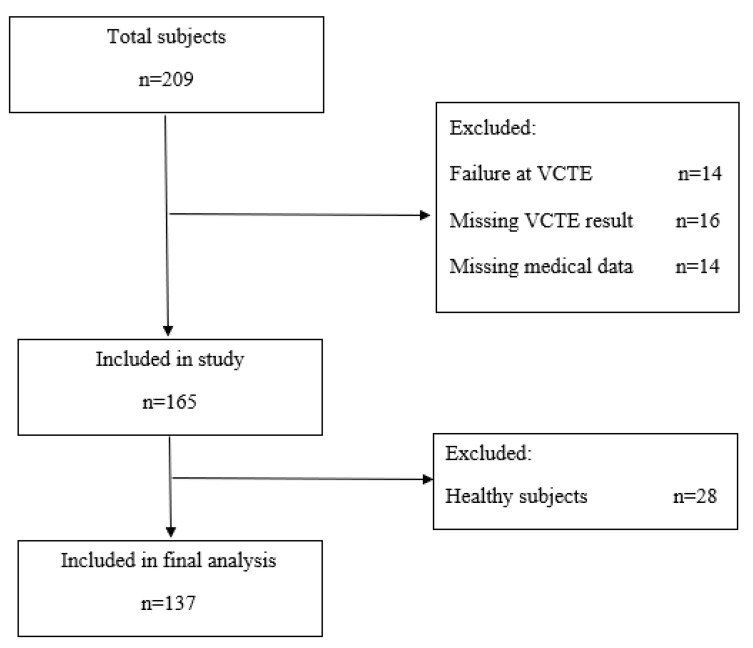
Study flow chart diagram.

Two hundred and nine patients were scanned using the THE system. Forty-four patients were excluded from the analysis, due to failure at LSMs by VCTE, missing medical data and laboratory data from medical records, and for not having undergone LS measurements by both elastography methods (Figure 3). One hundred sixty-five patients were included the final analysis. The patients’ main characteristics are reported in Table 1. 

Unreliable or failure values at LSMs were observed in 14/209 patients (6.6%) patients by VCTE (4 of them had ascites) and in zero patients by THE. The unreliable results were due to obesity, *p* < 0.0001. The M probe at VCTE was used in 125 patients (75.5%) and the XL probe in 40 patients (24.5%), *p* < 0.0001. 

The mean LS values in healthy subjects were significantly higher by THE than by VCTE: 6.94 ± 0.75 kPa vs. 5 ± 1.27 kPa, *p* < 0.0001. To avoid biases in evaluating the THE performance for liver fibrosis prediction, we excluded healthy subjects from further analysis 

A direct, significant and strong correlation (r = 0.82) was observed between LSM assessed by VCTE and THE, *p* < 0.0001. The mean values of liver stiffness for each fibrosis stage are reported in Table 2. The THE system had higher LSM values than TE in F0-F1 stages, but lower LSM values than VCTE in F2-F4 stages.

### 3.2. The Cut-Off Values for Predicting Different Stages ofLiver Fibrosis

**Figure 4:**
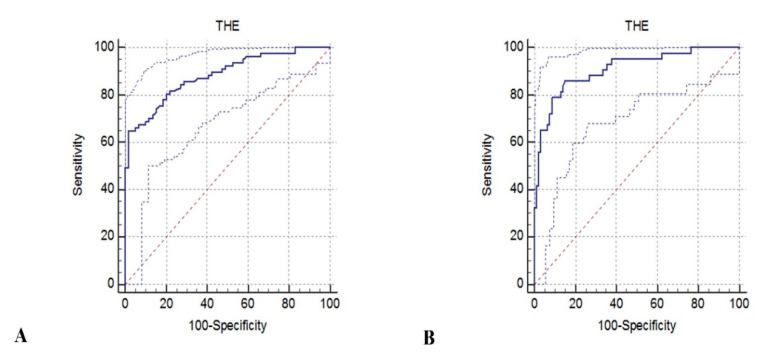
(**A**). Diagnosis performance of THE system for ≥ F2 (significant fibrosis) according to ROC analysis. (**B**). Diagnosis performance of THE system for F4 (liver cirrhosis) according to ROC analysis.

AUROCs (Figure 4) as well as diagnosing performance and optimal thresholds for significant fibrosis and liver cirrhosis are described in Table 3. Accuracy was the highest for F4 (liver cirrhosis), with an AUROC of 0.90 (95% CI 0.82–0.93) and dropped a little for F ≥ 2 (significant fibrosis). For significant fibrosis, the optimal cut-off value was 7.58 kPa, Se = 81.8%, Sp = 77.9%, NPV = 76.7%, PPV = 82.9%. For a better workflow, in clinical practice, we used the rule-in or rule-out term for differentiating advanced liver disease (ALD) (Table 3). 

### 3.3. Parameters That Influenced the THE Results.

Higher values of AST (*p* = 0.0004), ALP (*p* = 0.04), GGT (*p* = 0.002), age over 60 years old (*p* < 0.0001), and female gender (*p* < 0.0001) were factors that influenced the THE values in univariate analysis, but in multivariate analysis, none of the factors were independently associated with the THE values (Table 4). 

### 3.4. Intra and Interobserver Reproducibility of LS Measurements by the THE System

**Figure 5:**
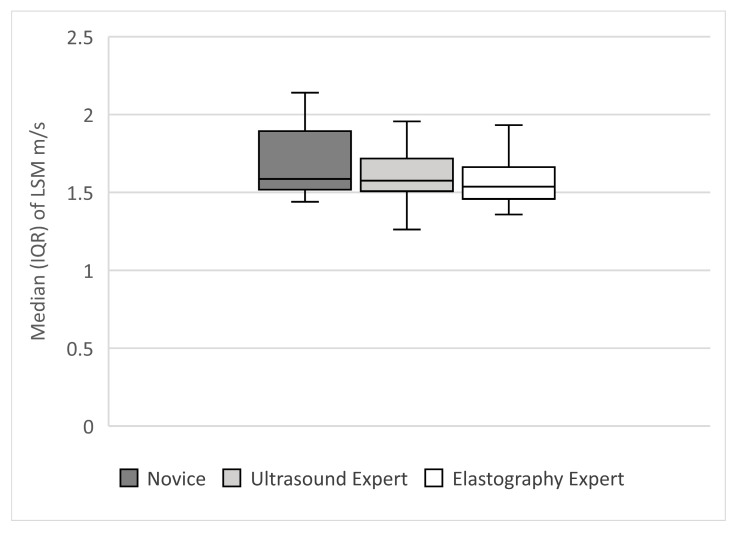
Elastographic measurements obtained by the three categories of examiners: novice, elastography expert and ultrasound expert. Boxplots depict 25–75% interquartile range (box), median (middle line), 5–95% range (two outer lines).

This sub-study included 27 subjects: 13 women and 14 men, with a mean age of 41 (range 19–75) years and mean BMI 25.00 ± 3.89 kg/m^2^. More than half (62.9%) were healthy volunteers and 37.1% had chronic hepatopathies. We did not find significant differences between the means of LSM obtained by different categories of examiners, overall and across the study group (1.66 ± 0.12 m/s vs. 1.66 ± 0.11 m/s vs. 1.65 ± 0.3 m/s, *p* = 0.76) (Figure 5). The overall agreement between examiners was excellent: 0.94 (95% CI: 0.89–0.97). The agreement between the novice and the experienced examiners, respectively, was good (novice and ultrasound expert: k = 0.80 (95% CI: 0.67–0.94); novice and elastography expert: k = 0.81 (95% CI: 0.69–0.94). The agreement between the ultrasound expert and elastography expert was good to excellent: k = 0.89 (95% CI: 0.82–0.96). 

The intra-observer reproducibility for each of the examiners was excellent, however the ICCs were higher for the more experienced elastographic examiner: 0.92, (95% CI: 0.82–0.96) vs. 0.94 (95% CI: 0.87–0.97) vs. 0.97 (95% CI: 0. 95–0.99). The concordance correlation coefficients were similar: novice vs. ultrasound expert: 0.84; novice vs. elastography expert: 0.89 and ultrasound expert vs. elastography expert: 0.89.

## 4. Discussions

THE is an ultrasound-based time-harmonic multifrequency shear wave elastographic method used to measure stiffness tissue and wave speed dispersion. It comes with a few advantages as compared with other elastographic methods: scanning of the entire ultrasound section-view; it is a good method to evaluate the viscoelastic properties of the liver; there are no limitations such as obesity and ascites; and the costs are lower. 

There are previous studies which evaluated the THE method, such as the study of Tzschätzsch et al. [9], where the method’s feasibility in measuring the viscoelastic response of the liver was assessed. A direct comparison was made between the THE method and magnetic resonance elastography (MRE), used for validation, with similar results for the in vivo quantification of the shear viscoelastic properties of the liver, but with significantly lower costs and in real time. However, the study was performed only on eight healthy volunteers and one cirrhotic patient [9]. 

In another study, Selcan Ipek-Ugay [19] analyzed the sensitivity of THE when measuring liver stiffness changes following water and food intake. Even though transient elastography is not influenced by water intake [20], THE has the advantage of approaching the entire liver (up to 13 cm) which is excited by a multifrequency mechanical stimulus, sensing wider range of tissue dynamics, thus achieving higher sensitivity, even to the minimal physiological changes after fluid and food ingestion. 

**Figure 6:**
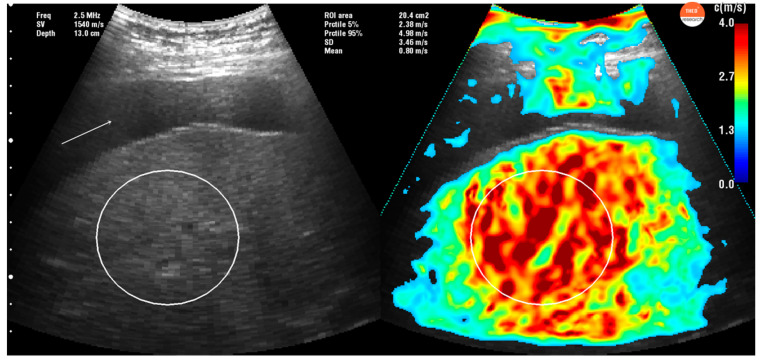
Liver stiffness measurement obtained with THE in a cirrhotic patient with ascites (white arrow). In this picture, we can see the acquisition of the LSM (in the right side of the figure) reflecting cirrhotic liver in orange-red. We can depict the orange-red appearance of the cirrhotic liver even if ascites is present. The current ROI has an area of 20.4 cm^2^ with a corresponding LS (mean ± SD, (5% percentile, 95% percentile)) of shear wave speed = 3.46 m/s ± 0.80 m/s (2.38 m/s, 4.98 m/s) and Youngs modulus = 35.91 kPa ± 16.61 kPa (16.99 kPa, 74.40 kPa) respectively.

Feasibility of VCTE is sometimes limited, mainly due to ascites and high BMI [5,6]. The results of our study show that the THE system is a reliable method for LS measurement, obtaining a feasible result in 100% of cases in comparison with VCTE where 9.6% of cases were considered failure, probably due to obesity (mean BMI = 27.4 kg/m^2^). Hudert et al. [21] prospectively assessed 67 pediatric obese patients with a mean BMI of 34.7 kg/m^2^ and biopsy proven non-alcoholic fatty liver disease (NAFLD). THE had 100% feasibility in that group of patients and the cutoffs calculated for different liver fibrosis stages were similar with ours: 1.52 m/s for F1, 1.62 m/s for F2, and 1.64 m/s for F3. Four patients from our cohort had ascites secondary to liver cirrhosis on ultrasound examination, and even if they were excluded from the study (failure at TE), THE managed to correctly evaluate these patients (Figure 6), suggesting the diagnosis of cirrhosis by the high values of LSM. 

Interestingly, according to our results, LSM values obtained by THE system were higher than those obtained by TE for F1-F0 patients and lower than those obtained by TE for F2-F4 patients. We did not find any explanation for these results, probably the small number of patients in each category of fibrosis is a cause of bias worth taking into consideration. Nevertheless, liver inflammation as an ingredient of liver stiffness may play a role in solving this question. Acknowledging the results form univariate analysis where AST was one of the parameters influencing THE’s results we can assume that inflammation could alter the results, nevertheless this idea should be backed up by further, stronger studies. 

AUROC curve was highest for F4 0.90 (95% CI 0.82–0.93), highlighting the value of the method in diagnosis liver cirrhosis. If we compare it with other elastographic methods such as pSWE (point shear wave-elastography) using ARFI (acoustic radiation force imaging) in patients with liver cirrhosis with AUROCs of 92–93% [22,23,24], we notice similar results. 

In a sub-group of 27 patients, we also analyzed the intra- and interobserver reproducibility of THE. The agreement between the three types of examiners (novice, elastography expert, and ultrasound expert) was excellent, demonstrating a good inter-observer reproducibility. However, when talking about the intra-observer reproducibility, LSM performed by the experienced examiner were more homogeneous (higher ICCs) meaning that experience may play a role in acquiring reliable LSM by THE system. This observation was also made in another study [25] that evaluated the reproducibility of a 2D-SWE system, with similar results as ours in what regards the operator experience in performing the elastographic technique. 

This study has some limitations related both to the new device and the reference method. The examination time/patient is a few minutes longer than VCTE examination, with a standard THE evaluation lasting for about five minutes. The vibrations produced by the machine might be discomforting for some patients, especially if the examination requires extra time for the evaluator. Another drawback of the device would be the vibration device itself that is an extra feature of the system, requiring a special design bed, which increases the method’s cost. At the moment, THE does not support continuous operation due to system overheating. 

Another limitation of our study is the use of another elastography technique and not of liver biopsy as a reference method. But TE is accepted nowadays by the international guidelines as a reliable method for the noninvasive staging of liver fibrosis [5,6].

## 5. Conclusions

THE is a feasible elastography technique that can accurately rule-in or rule-out advanced liver disease. It turns out to be a reproducible and easy to learn method; ultrasound or elastographic experience not influencing the accuracy of LSMs. THE may become a noninvasive elastographic method for evaluating liver fibrosis with a better liver parenchymal approach. 

## Figures and Tables

**Table 1:** Patient demographics and biochemical tests.

Characteristics	*n* = 165
Age, years (mean ± SD)	53.5 ± 16.1
Gender, male (%)	96 (58.1%)
BMI, kg/m^2^ (mean ± SD)	27.4 ± 5.25
AST, IU/L (median, 25th pct–75th pct)	31 (6–78)
ALT, IU/L (median, 25th pct–75th pct)	30 (10–190)
GGT, IU/L (median, 25th pct–75th pct)	42.5 (20–800)
ALP, IU/L (median, 25th pct–75th pct)	80 (6.7–315)
Platelet count, 10^3^/mm^3^ (median, 25th pct–75th pct)	182 (25–574)
Fibrosis stage (assessed with VCTE) (%)	
(F0-F1)	82 (49.6%)
(F2)	26 (15.7%)
(F3)	11 (6.6%)
(F4)	46 (28.1%)
Etiology of chronic liver diseases (%)	
Hepatitis B virus (HBV)	35 (21.2%)
Hepatitis C virus (HCV)	46 (27.9%)
Non-alcoholic fatty liver disease (NAFLD)	31 (18.7%)
Alcohol liver disease	25 (15.2%)
Healthy subjects	28 (17%)

SD = standard deviation; IQR = interquartile range; BMI = body mass index; AST = aspartate aminotransferase; ALT = alanine aminotransferase; GGT = gamma-glutamyl transferase; ALP = alkaline phosphatase; VCTE = vibration controlled transient elastography.

**Table 2:** Mean values of liver stiffness for each fibrosis stage by THE and VCTE.

Fibrosis Stage (*n* = 137)	VCTE, kPa	THE, kPa	*p*-Value
F0-F1 (*n* = 54)	5.08 ± 1.01	7.05 ± 0.74	<0.0001
F2-F3 (*n* = 37)	8.89 ± 1.13	8.21 ± 1.07	0.006
F4 (*n* = 46)	26.57 ± 13.50	11.81 ± 3.02	<0.0001

**Table 3:** Diagnosing performance of LSM evaluated by means of THE for various stages of fibrosis, using VCTE as reference the method.

		F0-1 vs. F2-4	F0-3 vs. F4
**AUROC (95% CI)**		0.88 (0.65–0.94)	0.90 (0.82–0.93)
***p*-value**		<0.0001	<0.0001
**Youden criteria**	Cut-off	1.59 m/s (7.58 kPa)	1.75 m/s (9.18 kPa)
	Se (95% CI)	81.8% (71.4–89.7%)	79% (64–90%)
	Sp (95% CI)	77.9% (65.3–87.7%)	91.4% (83.9–96.2%)
	PPV (95% CI)	82.9% (72.5–89.7%)	81% (65.9–91.4%)
	NPV (95% CI)	76.7% (64–86.6%)	90.4% (82.6–95.5%)
	LR+ (95% CI)	3.71 (2.3–6.1)	9.19 (4.7–18.1)
	LR− (95% CI)	0.23 (0.1–0.4)	0.23 (0.1–0.4)
**Rule out** Se > 90%, NPV > 90%	Cut-off	1.49 m/s (6.66 kPa)	1.61 m/s (7.77 kPa)
	Se (95% CI)	97.4% (90.9–99.7%)	90.7% (77.9–97.4%)
	Sp (95% CI)	32.2% (20.6–45.6%)	66.6% (56.3–76.1%)
	PPV (95% CI)	65.8% (55.8–73.9%)	55.7% (43.3–67.6%)
	NPV (95% CI)	90.0% (69.6–98.9%)	93.9% (85.2–98.3%)
	LR+ (95% CI)	1.44 (1.2–1.7)	2.72 (2–3.7)
	LR− (95% CI)	0.08 (0.02–0.3)	0.17 (0.07–0.4)
**Rule in** Sp > 90%, PPV > 90%	Cut-off	1.65 m/s (8.16 kPa)	1.83 m/s (10.04 kPa)
	Se (95% CI)	67.5% (55.9–77.8%)	65.1% (49.1–79%)
	Sp (95% CI)	91.5% (81.3–97.2%)	96.7% (90.9–99.3%)
	PPV (95% CI)	91.2% (80.7–97.1%)	90.3% (74.2–98%)
	NPV (95% CI)	68.4% (57.4–78.7%)	85.7% (77.5–91.8%)
	LR+ (95% CI)	7.97 (3.4–18.7)	20.1 (6.5–62.8)
	LR− (95% CI)	0.35 (0.3–0.5)	0.36 (0.2–0.5)

Se = sensibility; Sp = specificity; NPV = negative predictive value; PPV = positive predictive value; LR− = negative likelihood ratio; LP+ = positive likelihood ratio; CI = confident interval; AUROC = aria under the operating characteristic curve.

**Table 4:** Univariate and multivariate analysis of factors associated with the THE values.

Parameter	Univariate Analysis *p*-Value	Multivariate Analysis *p*-Value
AST	0.0004	0.18
ALT	0.64	NA
BMI	0.88	NA
AGE > 60 years	<0.0001	0.1
ALP	0.04	0.27
GGT	0.002	0.56
Female gender	<0.0001	0.51
Platelets	0.54	NA

NA = not applicable.
